# Genetic variation in PPARGC1A may affect the role of diet-associated inflammation in colorectal carcinogenesis

**DOI:** 10.18632/oncotarget.14347

**Published:** 2016-12-29

**Authors:** Young Ae Cho, Jeonghee Lee, Jae Hwan Oh, Hee Jin Chang, Dae Kyung Sohn, Aesun Shin, Jeongseon Kim

**Affiliations:** ^1^ Molecular Epidemiology Branch, National Cancer Center, Goyang, South Korea; ^2^ Center for Colorectal Cancer, National Cancer Center Hospital, National Cancer Center, Goyang, South Korea; ^3^ Department of Preventive Medicine, Seoul National University College of Medicine, Seoul, South Korea

**Keywords:** diet, inflammation, polymorphism, PPARGC1A, interaction

## Abstract

The role of inflammation in colorectal carcinogenesis may differ according to individuals’ genetic variations. Therefore, we investigated whether genetic susceptibility alters the association between inflammatory potential of diet and the risk of colorectal cancer within the Korean population. We genotyped four polymorphisms in four genes (*IL1B*, *TNF*, *PPARG*, and *PPARGC1A*) and calculated the dietary inflammatory index (DII) in a case-control study with 701 colorectal cancer patients and 1,402 controls. Among the investigated polymorphisms, heterozygous carriers of rs3774921 in *PPARGC1A* showed a higher risk of colorectal cancer (OR [95% CI] = 1.26 [1.02–1.55] for TC vs. TT). When the data were stratified by rs3774921 genetic variant, the association of a pro-inflammatory diet with colorectal cancer risk was more prominent among homozygous variant allele carriers (OR [95% CI] = 5.15 [2.35–11.29] for high vs. low DII) (*P* for interaction = 0.009). When stratified by anatomic site, this association was much stronger for rectal cancer patients (OR [95% CI] = 8.06 [2.67–24.16] for high vs. low DII) (*P* for interaction = 0.006). Additionally, this interaction was stronger among those older than 50 years and not exercising regularly. Conversely, no association or interaction was found for the other investigated polymorphisms. In conclusion, the results of this study suggest that a pro-inflammatory diet may have a differential effect on colorectal cancer risk based on *PPARGC1A* genetic variation. This interaction may differ by anatomic location and other risk factors.

## INTRODUCTION

Chronic inflammation is known to play an important role in colorectal cancer [1], and certain dietary components (e.g. fruit and vegetables, omega-3 polyunsaturated fatty acid, vitamin D) may modulate inflammation [2]. Recently, the dietary inflammatory index (DII) was developed to evaluate the inflammatory potential of diet [3]. This measure is reported to be associated with both the level of inflammatory cytokines [4] and the risk of colorectal cancer [5]. In some Asian countries, recent change in dietary habits may elevate inflammation and partly contribute to the marked increase in the incidence of colorectal cancer [6].

The role of diet in inflammation and colorectal carcinogenesis can differ according to an individual's genetic susceptibility [7]. Several previous studies have suggested that inflammation-related genetic variants may be associated with risk of colorectal cancer [8, 9]. Interleukin 1-beta (IL1B) and tumor necrosis factor (TNF) are important proinflammatory cytokines involved in cell growth, differentiation, apoptosis, and carcinogenesis [10, 11]. Peroxisome proliferator-activated receptor gamma (PPARγ) regulates lipid and glucose metabolism [12]. PPARγ coactivator 1-alpha (PGC-1α), which interact with PPARγ, is a transcriptional coactivator that has important functions in energy metabolism [13]. Both PPARγ and PGC-1α are reported to exert anti-inflammatory effects by reducing the circulating levels of proteins that serve as inflammatory markers [12, 14]. In addition, certain polymorphisms in genes regulating these proteins are reported to be associated with colorectal cancer [15–18]. Although growing evidence emphasizes the role of inflammation in colorectal carcinogenesis, studies investigating interaction between inflammation-related polymorphisms and diet are still lacking [7, 19].

In our previous study, we found that pro-inflammatory diet was associated with the increased risk of colorectal cancer in a Korean population [20]. Therefore, in this study, we aimed to investigate whether genetic susceptibility alters the role of diet-associated inflammation in colorectal carcinogenesis. We selected four genetic polymorphisms in four genes that are involved in inflammation and colorectal cancer risk [15–18]. We also examined whether this interaction differs according to anatomic location and other risk factors.

## RESULTS

The characteristics of the controls and cases are presented in Table 1. Compared to the controls, the cases were more likely to have a family history of colorectal cancer (*P* < 0.001), to not be highly educated (*P* < 0.001), and to have a low level of regular exercise (*P* < 0.001). The cases showed higher total caloric intake (*P* < 0.001) and DII score (*P* < 0.001) compared to the controls. In contrast, there were no significant differences between the cases and controls in terms of body mass index (BMI), smoking status and alcohol consumption. A higher DII score (representing a more pro-inflammatory diet) was associated with an increased incidence of colorectal cancer (OR [95% CI] = 1.78 [1.45–2.18] for high vs. low). Rectal cancer showed stronger associations compared to colon cancer (See Supplementary Table 1).

**Table 1:** General characteristics of the study subjects^a^

	Controls (n=1402)	Cases (n=701)	*P*-value
**Age (years)**	56.0±9.1	56.4±9.6	0.31
**Female**	444(31.7)	222(31.7)	>0.99
**Family history of colorectal cancer^b^**	77(5.5)	69(9.8)	**<0.001**
**BMI (kg/m^2^)**			
<25	935(66.7)	479(68.3)	0.45
≥25	467(33.3)	222(31.7)	
**Education level**			
Middle school or less	197(14.2)	254(36.2)	**<0.001**
High school	455(32.7)	269(38.4)	
College or more	739(53.1)	178(25.4)	
**Smoking status**			
Nonsmoker	615(43.9)	315(44.9)	0.64
Ever smoker	787(56.1)	386(55.1)	
**Alcohol consumption**			
Nondrinker	420(23.0)	211(30.1)	0.95
Ever drinker	982(70.0)	490(69.9)	
**Regular exercise (yes)**	830(59.5)	229(32.7)	**<0.001**
**Total caloric intake (kcal/day)**	1698.5±560.5	2020.1±530.0	**<0.001**
**Sum of DII**	1.07±2.25	1.87±1.96	**<0.001**

Abbreviation: BMI, body mass index; DII, dietary inflammatory index.

^a^Results are presented as the mean ± SD or n (%).

^b^First-degree relative.

The basic information of the investigated polymorphisms is shown in Table 2. The minor allele frequencies of the controls for all four single-nucleotide polymorphisms (SNPs) were as follows: rs4848306 (0.48), rs1800629 (0.08), rs1801282 (0.06), and rs3774921 (0.32). In addition, all SNPs in the control subjects were in Hardy-Weinberg equilibrium. Among the investigated polymorphisms, an association with a higher risk of colorectal cancer was observed for heterozygous carriers of rs3774921 in the *PPARGC1A* (OR [95% CI] = 1.26 [1.02–1.55] for TC vs. TT). However, the other investigated polymorphisms did not show any association with colorectal cancer (Table 3).

**Table 2:** Primary information for the four polymorphisms included in this study^a^

Gene	Ch. location	rs number	Base change	Functional consequence	MAF^b^	Ref
***IL1B***	2q14	rs4848306	G>A	upstream variant	0.48	[15]
***TNF***	6p21.3	rs1800629	G>A	upstream variant	0.08	[16]
***PPARG***	3p25	rs1801282	C>G	missense, intron variant	0.06	[17]
***PPARGC1A***	4	rs3774921	T>C	intron variant	0.32	[18]

Abbreviations: Ch, chromosome; MAF, minor allele frequency.

^a^Data were obtained from the NCBI dbSNP database (http://www.ncbi.nlm.nih.gov/projects/SNP).

^b^Minor allele frequency in the controls of this study.

**Table 3:** Association between inflammation-related genetic polymorphisms and the risk of colorectal cancer

	No. of Controls (%)	Colorectal Cancer		Colon Cancer		Rectal Cancer	
		No. of Cases (%)	Adjusted OR (95% CI)^a^	No. of Cases (%)	Adjusted OR (95% CI)^a^	No. of Cases (%)	Adjusted OR (95% CI)^a^
***IL1B*** **rs4848306**							
GG	393(27.2)	209(30.1)	1.0(ref)	104(29.6)	1.0(ref)	102(31.0)	1.0(ref)
GA	695(49.8)	339(48.9)	1.06(0.81–1.27)	169(48.0)	0.98(0.74–1.29)	165(50.2)	1.09(0.82–1.45)
AA	307(22.0)	146(21.0)	0.98(0.74–1.31)	79(22.4)	0.90(0.63–1.29)	62(18.8)	1.11(0.77–1.60)
***TNF*** **rs1800629**							
GG	1192(85.1)	598(86.0)	1.0(ref)	302(85.8)	1.0(ref)	284(85.8)	1.0(ref)
GA	203(14.5)	90(13.0)	0.88(0.66–1.17)	46(13.1)	0.89(0.62–1.28)	44(13.3)	0.90(0.62–1.30)
AA	5(0.4)	7(1.0)	2.03(0.60–6.89)	4(1.1)	2.30(0.58–9.14)	3(0.9)	1.82(0.41–8.14)
***PPARG*** **rs1801282**							
CC	1239(89.1)	626(89.4)	1.0(ref)	323(91.2)	1.0(ref)	291(87.4)	1.0(ref)
CG	143(10.7)	72(10.3)	0.84(0.61–1.17)	30(8.5)	0.69(0.45–1.07)	41(12.3)	1.02(0.69–1.52)
GG	2(0.1)	2(0.3)	2.99(0.38–23.33)	1(0.3)	2.80(0.24–33.25)	1(0.3)	3.30(0.28–39.42)
***PPARGC1A*** **rs3774921**							
TT	679(48.5)	316(45.3)	1.0(ref)	153(43.5)	1.0(ref)	159(47.9)	1.0(ref)
TC	567(40.5)	316(45.3)	**1.26(1.02–1.55)**	170(48.3)	**1.40(1.08–1.81)**	139(41.9)	1.11(0.85–1.45)
CC	154(10.1)	65(9.3)	0.91(0.65–1.29)	29(8.2)	0.83(0.53–1.31)	34(10.2)	0.95(0.61–1.46)

^a^Adjusted for education and total calorie intake.

We found that the association between DII score and colorectal cancer risk differed according to the genetic variant of *PPARGC1A* (Table 4). When the data were stratified by *PPARGC1A* genotype, the association of a pro-inflammatory diet with colorectal cancer risk was significantly more pronounced among those carrying rs3774921 homozygous variant allele (OR [95% CI] = 5.15 [2.35–11.29] for high vs. low DII); the association for the wild-type allele was much weaker (OR [95% CI] = 1.63 [1.31–2.02] for high vs. low DII) (*P* for interaction = 0.009). When stratified by anatomic site, this association was much stronger in rectal cancer patients (OR [95% CI] = 8.06 [2.67–24.16] for high vs. low DII) (*P* for interaction = 0.006) than in colon cancer patients (OR [95% CI] = 3.33 [1.24–8.93] for high vs. low DII) (*P* for interaction = 0.26) carrying rs3774921 homozygous variant allele. When we examined the combined effect of this SNP and the DII score, the association of this genetic variant with colorectal cancer risk differed according to the DII score. Compared with wild-type carriers, those harboring the homozygous variant showed a decreased risk of colorectal cancer among those with a low inflammatory diet (OR [95% CI] = 0.46 [0.26–0.82] for CC vs. TT/CT) but an increased risk among those with a high inflammatory diet (OR [95% CI] = 1.90 [1.23–2.94] for CC vs. TT/CT). This observed interaction was much stronger among rectal cancer patients (Table 4; See Supplementary Figure 1). Conversely, no interaction with DII score in relation to colorectal cancer risk was found for the other investigated polymorphisms (data not shown).

**Table 4:** Association of DII score with the risk of colorectal cancer, colon cancer, and rectal cancer, stratified by *PPARGC1A* rs3774921 genetic variant^a^

DII score		Combined effect By SNP and DII		Main effect of DII by the SNP	*P* for interaction
		Low	High	High vs. Low	
**Colorectal cancer**					
TT/TC	No.Controls/Cases	616/246	630/386		
	OR (95% CI)^b^	1.0 (ref)	**1.61(1.30, 2.00)**	**1.63(1.31, 2.02)**	**0.009**
CC	No.Controls/Cases	83/17	71/48		
	OR (95% CI)^b^	**0.46(0.26, 0.82)**	**1.90(1.23, 2.94)**	**5.15(2.35, 11.29)**	
**Colon cancer**					
TT/TC	No.Controls/Cases	616/130	630/193		
	OR (95% CI)^b^	1.0 (ref)	**1.53(1.17, 2.00)**	**1.55(1.19, 2.03)**	0.26
CC	No.Controls/Cases	83/11	71/18		
	OR (95% CI)^b^	0.57(0.29, 1.12)	1.45(0.81, 2.61)	**3.33(1.24, 8.93)**	
**Rectal cancer**					
TT/TC	No.Controls/Cases	616/113	630/185		
	OR (95% CI)^b^	1.0 (ref)	**1.68(1.27, 2.22)**	**1.68(1.27, 2.23)**	**0.006**
CC	No.Controls/Cases	83/6	71/28		
	OR (95% CI)^b^	**0.37(0.15, 0.90)**	**2.41(1.43, 4.06)**	**8.06(2.67, 24.16)**	

Abbreviation: DII, dietary inflammatory index; SNP, single nucleotide polymorphism.

^a^The DII score was categorized into two groups (high/low) based on the median (1.41) level of the control group.

^b^Adjusted for education and total calorie intake.

We also examined whether the interaction between *PPARGC1A* genetic variation and diet-associated inflammation in relation to colorectal cancer risk could be modified by other risk factors such as age, BMI, regular exercise, and smoking status. Interaction between the *PPARGC1A* rs3774921 genotype and DII score in relation to colorectal cancer risk was much stronger among those who were older than 50 years (*P* for interaction = 0.005), were not overweight (*P* for interaction = 0.03), and did not exercise regularly (*P* for interaction = 0.004) (Table 5).

**Table 5:** Association of DII score with the risk of colorectal cancer, as stratified by *PPARGCA1* rs3774921 genetic variant and risk factors^a^

DII score	TT/TC			CC			*P* for interaction
	No. Controls/Cases		High vs. Low	No. Controls/Cases		High vs. Low	
	Low	High	Adjusted OR (95% CI)^b^	Low	High	Adjusted OR (95% CI)^b^	
**Age**							
<50 years old	116/63	162/82	0.95(0.62, 1.45)	14/4	21/8	0.86(0.16, 4.72)	0.83
≥50 years old	500/183	468/304	**1.96(1.50, 2.48)**	69/13	50/40	**8.42(3.24, 21.90)**	**0.005**
**BMI (kg/m^2^)**							
<25	402/160	429/270	**1.77(1.36, 2.32)**	54/10	50/35	**6.63(2.33, 18.87)**	**0.03**
≥25	214/86	201/116	1.40(0.97, 2.02)	29/7	21/13	**3.81(1.09, 13.28)**	0.15
**Regular exercise**							
No	202/171	308/257	1.03(0.77–1.38)	25/9	30/33	**8.54(2.27–32.13)**	**0.004**
Yes	410/75	320/129	**2.47(1.75–3.50)**	57/8	41/15	2.76(0.94–8.18)	0.75
**Smoking status**							
Never	301/121	248/163	**1.91(1.39–2.63)**	36/6	30/22	**7.16(1.93–26.60)**	0.06
Ever	315/125	382/223	**1.46(1.09–1.96)**	47/11	41/26	**4.91(1.71–14.13)**	0.08

Abbreviation: DII, dietary inflammatory index.

^a^The DII score was categorized into two groups (high/low) based on the median (1.41) level of the control group.

^b^Adjusted for education and total calorie intake.

## DISCUSSION

The present study suggests that in a Korean population, the association between the inflammatory potential of diet and colorectal cancer risk may differ according to genetic variations in *PPARGC1A*. These interactions were stronger among rectal cancer patients compared to those with colon cancer.

Several studies have reported the possible association between genetic variations in inflammation pathway genes and colorectal cancer [8, 9]. Among the polymorphisms investigated in the present study, only the *PPARGC1A* variant appears to be associated with the risk of colorectal cancer. PGC1α is a major regulator of several key metabolic pathways, including glucose, lipid, cellular energy metabolism as well as reactive oxygen species (ROS) defense system pathways [13]. Although there are conflicting data regarding the role of PGC1α in carcinogenesis, some studies have suggested a protective role against colorectal cancer by showing that PGC1α induces apoptosis in colorectal cancer cells and prevents tumor formation [21]. *PPARGC1A* was also recently reported to exert an anti-inflammatory effect [14, 22]. *In vivo*, mice lacking a functional *PPARGC1A* allele in muscle tissue present significant increases in the expression and release of pro-inflammatory factors [14, 23]. It has been suggested that *PPARGC1A* suppresses ROS production by mediating expression of genes regulating ROS detoxification and uncoupling proteins that reduce ROS production [24, 25]. Because ROS induce pro-inflammatory cytokine production in skeletal muscle [26], decreased expression of anti-ROS genes in the muscle-specific *PPARGC1A* knockout may have contributed to increases in cytokine expression. Additionally, *PPARGC1A* may directly affect expression of genes with either pro- or anti-inflammatory functions [22], and PGC1α is also suggested to inhibit NF-kB activity in muscle [27]. In the present study, carriers of a particular *PPARGC1A* genetic variant exhibited an increased risk of colorectal cancer. It can be assumed that carriers of this *PPARGC1A* variant, with reduced *PPARGC1A* expression compared to noncarriers, may experience elevated levels of inflammation and thus an increased risk of colorectal cancer.

Both genetic and environmental factors may affect colorectal cancer risk [7]. Recent epidemiological studies utilizing DII scores have reported consistent associations of a pro-inflammatory diet with increased risk of colorectal cancer [5, 19, 28, 29], similar to our finding. As the DII focuses on the inflammatory potential of diet, it has some advantages over other methods that investigate the role of a single food or nutrient in disease etiology [3]. In our study, we observed a significantly stronger association for a pro-inflammatory diet with the risk of colorectal cancer among those harboring the rs3774921 homozygous variant allele of *PPARGC1A* compared to those carrying the wild-type allele. Interestingly, the homozygous variant allele was associated with a reduced risk of colorectal cancer compared to the wild-type allele in those with a low inflammatory diet, but risk increased sharply among those with a high inflammatory diet. Similar results were observed in a case-control study conducted in Spain [19] in which individuals carrying the *IL4* rs2243250 variant allele had a significantly increased risk of colorectal cancer when consuming a pro-inflammatory diet. It can be assumed that the inverse association of the *PPARGC1A* variant allele with colorectal cancer risk might be due to impacts on other anti-carcinogenic and anti-inflammatory pathways in individuals with a low inflammatory diet. In contrast, a high inflammatory diet may induce inflammation, thus resulting in a synergic increase in colorectal cancer risk in carriers of the variant allele [19]. Because the protein product modulates glucose, lipid and energy metabolism, *PPARGC1A* is known to be highly responsive to environmental stimuli and nutritional status [30], and abnormal *PPARGC1A* expression might cause metabolic problems, allowing certain cells to thrive in a particular environment such as the carcinogenic milieu [30]. However, to the best of our knowledge, it remains unknown how this intronic variant, rs3774921, alters the function of *PPARGC1A*. Overall, the number of functional intronic polymorphisms identified is increasing, and this variant may influence gene expression by affecting either the transcriptional activity or splicing efficiency of the gene [31]. It can be hypothesized that this SNP alters PGC1α activity in a manner that influences only a part of the multiple processes regulated by this protein [25]. Therefore, further experimental studies are required to elucidate the function of this SNP.

Because colorectal cancer is a heterogeneous disease, interaction between genetic variants and diet-associated inflammation may differ according to anatomic site. Several meta-analyses have reported that inflammatory markers such as IL6 and CRP are associated with an increased risk of colon cancer but not of rectal cancer [32, 33]. These differential associations likely reflect varying susceptibilities to inflammation of the colon and rectum [33]. Indeed, such differences between the colon and rectum with regard to metabolizing enzyme activity, physiological function, fecal composition, bile acid metabolism, and intestinal transit time may influence one's susceptibility to environmental factors [34]. In the present study, interaction between diet-associated inflammation and a *PPARGC1A* genetic variant was slightly stronger among those with rectal cancer. Further work is needed to determine how inflammation and metabolism contribute differently to carcinogenesis in the colon and rectum. In addition to anatomic site, other factors may affect the interaction between diet-associated inflammation and a *PPARGC1A* genetic variant in colorectal carcinogenesis. For example, this interaction was more significant among those older than 50 years and those who did not exercise regularly. Aging as well as physical inactivity and other unhealthy lifestyles can initiate DNA damage, leading to inflammation [25]. We speculate that increased inflammation combined with genetic risk factors may elevate the risk of colorectal cancer. These findings may allow more effective diet and lifestyle interventions in efforts to prevent colorectal cancer.

To the best of our knowledge, this is the first report of the interaction between diet-associated inflammation and a *PPARGC1A* genetic variant in relation to colorectal cancer. However, the present study has several limitations that should be considered. First, this study is a case-control study. The controls were selected among those who voluntarily participated in a health check-up program, thus they may have been more health conscious than the general population. Additionally the cases and controls may have differed with regard to their recall of dietary habits. However, the data were gathered using a validated questionnaire without knowledge of the specific hypotheses of this study. In addition, the cases consisted of newly diagnosed colorectal cancer patients, and the assessment of dietary intake was conducted before cancer diagnosis, thereby reducing the potential for differential misclassification and measurement errors. Second, only one SNP in each inflammation-related gene was evaluated, which may not represent the entire gene. Third, our sample size was relatively small to conduct the subgroup analyses by anatomic site and risk factors, thus did not have sufficient power to detect a small interaction effect.

In conclusion, a pro-inflammatory diet may increase the risk of colorectal cancer, particularly among those with genetic variation in the *PPARGC1A* gene. This study provides valuable insight into the underlying mechanisms by which diet-associated inflammation promotes colorectal carcinogenesis, and the findings suggest that inflammation may be linked to metabolic pathways. However, larger studies are required to validate the findings.

## MATERIALS AND METHODS

### Study population

Cases of colorectal cancer were recruited from the Center for Colorectal Cancer of the National Cancer Center, Korea, between August 2010 and August 2013. Among the 1,070 patients who agreed to participate in the study, 369 were excluded because of incomplete food frequency questionnaires (FFQs), missing blood samples, and implausible energy intakes. Therefore, 701 patients were included in the analysis. The control subjects were recruited between October 2007 and December 2014 among individuals visiting the Center for Cancer Prevention and Detection at the same hospital for a health check-up program provided by the National Health Insurance Cooperation, which covers the entire Korean population. Of the 14,201 individuals who agreed to participate in the study, 8,296 were excluded because of incomplete FFQs and questionnaires, implausible energy intakes, missing blood sample, and no agreement with regard to gene testing. Of the remaining 5,905 subjects, two controls per case were frequency matched by gender and 5-year age groups. Ultimately, 701 colorectal cancer patients and 1,402 healthy controls were selected for the final analysis (Supplementary Figure 2).

All participants provided written informed consent, and the study protocol was approved by the Institutional Review Board of the National Cancer Center (IRB No. NCCNCS-10-350 and NCC2015-0202).

### Data collection

Information on the participants’ (both cases and controls) demographic and lifestyle risk factors was collected using a structured questionnaire at initial recruitment, prior to cancer diagnosis. Each participant's habitual dietary intake was assessed using a 106-item FFQ; the validity and reproducibility of the questionnaire have been previously reported [35]. The participants provided their individual average frequency of eating and typical portion sizes in the year preceding the interview. These values were converted to obtain daily nutrient intake values using a scale with nine frequency categories (never or rarely, once a month, twice or three times a month, once or twice a week, three or four times a week, five or six times a week, once a day, twice a day, and three times a day) and three portion size categories (small, medium, and large). FFQ-derived data were used to calculate DII scores for all participants. Details of the development [3] and validation [4] of the DII have been previously described. To calculate the DII, we used the method previously reported by Shivappa et al.[3]. To calculate the DII score, this study included the following 36 food items: protein, fat, carbohydrate, fiber, monounsaturated fatty acid, saturated fatty acid, polyunsaturated fatty acid, n-3 fatty acid, n-6 fatty acid, cholesterol, thiamin, riboflavin, niacin, vitamin B_6_, vitamin B_12_, vitamin C, folic acid, vitamin A, vitamin D, vitamin E, β-carotene, iron, magnesium, selenium, zinc, ethanol, garlic, ginger, onion, green tea, flavan-3-ol, flavone, flavonol, flavanone, anthocyanidin, and isoflavone. Energy-adjustment was achieved using the residual method [36].

### SNP selection and genotyping

For this study, we selected single-nucleotide polymorphisms (SNPs) within *IL1B, TNF, PPARG*, and *PPARGC1*A. Based on a literature search, we selected four SNPs which were reported to be associated with colorectal cancer risk: rs4848306 (*IL1B*) [15], rs1800629 (*TNF*) [16], rs1801282 (*PPARG*) [17] and rs3774921 (*PPARGC1A*) [18].

The SNPs were genotyped as follows. Genomic DNA was extracted using the MagAttract DNA Blood M48 kit (Qiagen, Valencia, CA) and BioRobot M48 automatic extraction equipment (Qiagen) according to the manufacturers’ instructions. Genotyping was performed using the MassArray iPLEX® gold assay (Agena Bioscience, San Diego, CA), and primers were designed using the assay software. Up to 36 multiplex PCRs were performed using the iPLEX gold reagent and 20 ng of genomic DNA per reaction. The raw data were analyzed using TYPER ver 4.0 (Agena Bioscience). The call rate of all selected SNPs was over 95%.

### Statistical analyses

Differences in demographic and lifestyle factors between the cases and controls were analyzed using the chi-square test for categorical variables and t-tests for continuous variables. The DII score was categorized into two levels (high/low) based on median intake levels in the control group. Chi-square tests were used to test for Hardy-Weinberg equilibrium (HWE) for all SNPs in the control group. Crude and multivariable logistic regressions were applied to estimate the odds ratios (ORs) and 95% confidence intervals (CIs) of the associations of genetic polymorphisms and DII score with colorectal cancer risk. To decide which variables to enter into the multivariable model, we performed the backward selection using colorectal risk factors, which were selected based on both our data and prior information [37]. Finally, the multivariable model was adjusted for education and total caloric intake. A polytomous logistic regression model was used for subgroup analyses by anatomic location (colon/rectum).

To investigate gene-diet interaction, we examined the relationship between DII score (high/low) and colorectal cancer risk according to SNPs in inflammatory genes. To increase the statistical power, respective genotypes were combined into two groups; dominant (carriers of the rare allele vs. homozygotes for the common allele) or recessive (homozygotes for the rare allele vs. all others). Based on our power calculation, we selected a dominant model for all four SNPs and a recessive model for two SNPs with higher minor allele frequency (rs4848306 and rs3774921) [38]. We examined (i) the main effect of diet-associated inflammation in the strata defined by SNPs and (ii) the combined effect of both SNP and the DII. Interactions between DII scores and genetic polymorphisms were assessed using the likelihood ratio test by comparing the model with the interaction term, with the model containing only main effects. In addition, we examined whether the identified interaction between the genetic variant and DII score in relation to colorectal cancer risk is modulated by other risk factors by stratifying the data by age group (< 50 years old and ≥ 50 years old), BMI (< 25 kg/m^2^ and ≥ 25 kg/m^2^), regular exercise (yes/no), and smoking status (ever/never).

All statistical analyses were performed using SAS 9.2 software (SAS Institute Inc., Cary, NC). A two-sided *P*-value of less than 0.05 was considered statistically significant. Power analyses were conducted using Quanto 1.2.4 (http://biostats.usc.edu/Quanto.html; University of Southern California, Los Angeles).

## SUPPLEMENTARY MATERIALS FIGURES AND TABLES


