# Supplementary material for: Genetic variation in PPARGC1A may affect the role of diet-associated inflammation in colorectal carcinogenesis
Source: Oncotarget. 2016 Dec 29;8(5):8550–8. doi: 10.18632/oncotarget.14347 (PMC5352421; doi:10.18632/oncotarget.14347)
Supplement: Supplementary file 1 [file oncotarget-08-8550-s001.pdf]

Genetic variation in *PPARGC1A* may affect the role of diet-associated inflammation in colorectal carcinogenesis

SUPPLEMENTARY FIGURES AND TABLE

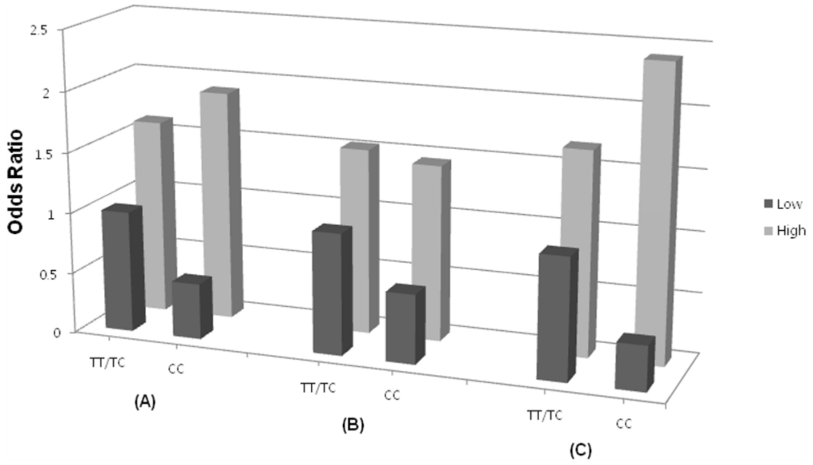

Supplementary Figure 1: Thecombined effects of DII score and genetic variants in *PPARGC1A* rs3774921 on the risk of colorectal A. colon **B.** and rectal cancer **C.** Wild-type carriers consuming a low inflammatory diet were considered as a reference. The DII score was categorized into two groups (high/low) based on median levels in the control group. The multivariable model was used by adjusting for education and total calorie intake.

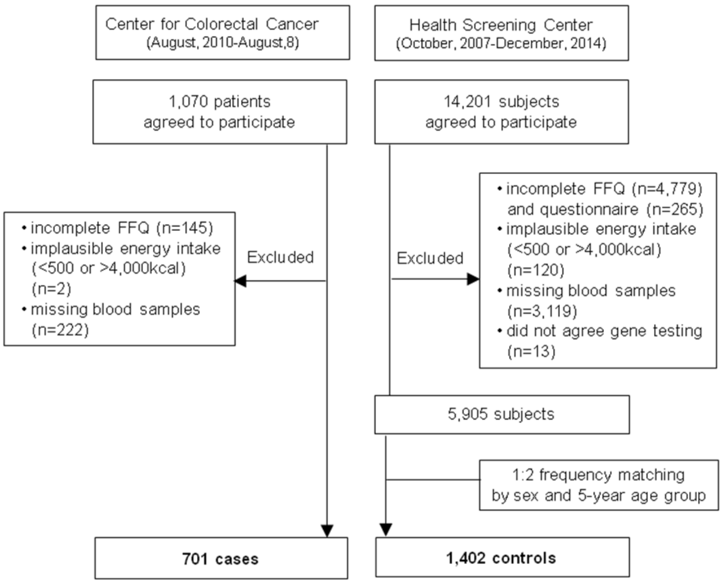

Supplementary Figure 2: Flow diagram of the selection of cases and controls.

Supplementary Table 1: Association between DII score and colorectal cancer risk

DII score[a]	No. of Controls (%)	No. of Cases (%)	Crude OR (95% CI)	Adjusted OR 95% CI)[b]
Colorectal cancer				
Low	701(50.0)	266(38.0)	1.0(ref)	1.0(ref)
High	701(50.0)	435(62.1)	1.64(1.36–1.97)	1.78(1.45–2.18)
Colon cancer				
Low	701(50.0)	142(40.1)	1.0(ref)	1.0(ref)
High	701(50.0)	212(59.9)	1.49(1.18–1.89)	1.62(1.26–2.08)
Rectal cancer				
Low	701(50.0)	121(36.2)	1.0(ref)	1.0(ref)
High	701(50.0)	213(63.8)	1.76(1.38–2.25)	1.92(1.48–2.50)

Abbreviation: DII, dietary inflammatory index.

[a]The DII score was categorized into two groups (high/low) based on the median (1.41) intake level of the control group.

[b]Adjusted for education and total calorie intake.
